# Gender Differences in the Relationship Between Coping Style and Attitudes Towards Palliative Care

**DOI:** 10.1093/geroni/igab046.3229

**Published:** 2021-12-17

**Authors:** Ilana Engel, Amber Watts, Tamara Baker, Christian Sinclair

**Affiliations:** 1 University of Kansas, Kansas. City, Missouri, United States; 2 University of Kansas, Lawrence, Kansas, United States; 3 University of North Carolina, Chapel Hill School of Medicine, University of North Carolina at Chapel Hill (School of Medicine), North Carolina, United States; 4 University of Kansas Medical Center, University of Kansas Medical Center, Kansas, United States

## Abstract

Palliative care (PC) is becoming more widely available and its benefits, including improved quality of life for patients, have been demonstrated. Studies on patient-level barriers to PC access focus on knowledge and misperceptions. This study aimed to explore, among a community sample, whether more approach-focused coping styles may be associated with more positive attitudes towards PC and whether more avoidant coping styles are associated with more negative attitudes towards PC. Two linear regression analyses (an approach model and an avoidance model) were conducted to determine predictors of attitudes towards PC, controlling for potential confounds. The sample consisted of 87 community-dwelling adults ages 65+ (mean age=72.72 (5.88); 56.32% = women; 86.21% = White). In both models, more knowledge of PC was associated with more positive attitudes towards PC (β = .71, p<.01). Coping by engaging more social support was significantly associated with more positive attitudes towards PC (β = .54, p<.05). Results demonstrated a significant interaction (β = -1.24, p<.01) such that women who endorsed high levels of disengaged coping reported more favorable attitudes towards PC than men who endorsed high levels of disengaged coping. Results indicate the need for a tailored approach to PC education for patients and families. Men who often cope with a stressor via distraction, self-blame, denial, or giving up may be less receptive to acceptance of PC. Future research on educational interventions tailored for individuals with distinct coping styles may be beneficial, particularly for men who frequently rely on disengaged coping styles.

